# Role of Rho GTPase Interacting Proteins in Subcellular Compartments of Podocytes

**DOI:** 10.3390/ijms22073656

**Published:** 2021-04-01

**Authors:** Kana Asano-Matsuda, Sajida Ibrahim, Tomoko Takano, Jun Matsuda

**Affiliations:** 1 Division of Nephrology, McGill University Health Centre, 1001 Decarie, Montreal, QC H4A 3J1, Canada; sajida.ibrahim@mail.mcgill.ca (S.I.); tomoko.takano@mcgill.ca (T.T.); 2 Department of Nephrology, Osaka University Graduate School of Medicine, 2-2 D11, Yamada-oka, Suita, Osaka 565-0871, Japan

**Keywords:** Cdc42, Rac1, RhoA, Rho GTPase, podocyte

## Abstract

The first step of urine formation is the selective filtration of the plasma into the urinary space at the kidney structure called the glomerulus. The filtration barrier of the glomerulus allows blood cells and large proteins such as albumin to be retained while eliminating the waste products of the body. The filtration barrier consists of three layers: fenestrated endothelial cells, glomerular basement membrane, and podocytes. Podocytes are specialized epithelial cells featured by numerous, actin-based projections called foot processes. Proteins on the foot process membrane are connected to the well-organized intracellular actin network. The Rho family of small GTPases (Rho GTPases) act as intracellular molecular switches. They tightly regulate actin dynamics and subsequent diverse cellular functions such as adhesion, migration, and spreading. Previous studies using podocyte-specific transgenic or knockout animal models have established that Rho GTPases are crucial for the podocyte health and barrier function. However, little attention has been paid regarding subcellular locations where distinct Rho GTPases contribute to specific functions. In the current review, we discuss cellular events involving the prototypical Rho GTPases (RhoA, Rac1, and Cdc42) in podocytes, with particular focus on the subcellular compartments where the signaling events occur. We also provide our synthesized views of the current understanding and propose future research directions.

## 1. Introduction

The kidney glomerulus is a structure with entangled capillaries where the first step of urine formation occurs by filtering the plasma into the urinary space. The glomerulus has a permselective barrier function that prevents macromolecules including blood cells and proteins from leaking into the urine. The glomerular filtration barrier consists of three layers: fenestrated endothelial cells, glomerular basement membrane (GBM), and podocytes [1].

Podocytes are terminally differentiated epithelial cells featured by actin-based projections called foot processes [2,3]. Foot processes from adjacent podocytes tightly interdigitate and are connected by a membrane-like structure called the slit diaphragm [4,5]. Many of the foot process membrane proteins are connected to the well-organized intracellular actin network. This network undergoes substantial changes in proteinuric kidney disease, leading to profound morphological changes known as “foot process effacement” [2,3,5,6].

The Rho family of small GTPases (Rho GTPases) act as intracellular molecular switches and tightly regulate the actin cytoskeletal dynamics [7,8]. Active Rho GTPases (GTP-bound form) interact with their effectors, leading to the activation of the downstream signaling pathways, which regulate actin networks and diverse cellular functions such as adhesion, migration, and spreading. Among 20 family members, RhoA, Rac1, and Cdc42 are the prototypical Rho GTPases and best studied in podocytes, while there are virtually no studies about the role of remaining 17 Rho GTPases. Rac1 and Cdc42 are best known in their role in the formation of the branched actin protrusions (lamellipodia) and thin bundles (filopodia), respectively, whereas RhoA typically facilitates myosin-decorated stress fiber formation [7].

A series of studies using podocyte-specific Rho GTPase transgenic or knockout (KO) animal models established that maintaining delicate balance of Rho GTPase activities is crucial for the podocyte health and barrier function. The most striking phenotype was observed in podocyte-specific Cdc42 KO mice which developed congenital nephrotic syndrome and died at around 3 weeks of age [9,10]. Inducible overexpression of dominant negative RhoA, constitutively active RhoA, or Rac1 in podocytes in mice caused foot process effacement with proteinuria [11,12,13,14]. Gene deletion of Rac1 in podocytes in mice caused no discernible basal renal phenotype. However, podocyte-specific Rac1 KO mice were protected in a protamine sulfate model of acute injury, while more susceptible in a chronic hypertensive glomerular injury model [10]. Several reviews are available, including ours, that provide the overview of the role of Rho GTPases in podocytes [15,16,17]. These reviews discuss the known roles of Rho GTPases and their regulatory proteins based on in vitro/in vivo studies and disease-causing variants in humans. Some recent studies have shown that targeting either Rho GTPases or their signaling pathways has an effect of preventing podocytopathy in mouse models. However, little attention has been paid regarding subcellular locations where distinct Rho GTPases contribute to specific functions.

**Figure 1:**
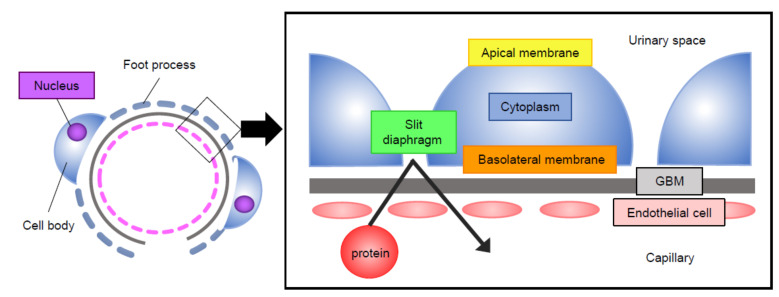
Podocytes in the kidney glomerulus. Glomerular filtration barrier consists of endothelial cells, glomerular basement membrane (GBM), and podocytes. Five compartments of podocytes (apical membrane, slit diaphragm, basolateral membrane, cytoplasm of the foot processes, and nucleus) are shown.

In the current review, we discuss cellular events involving Rho GTPases in podocytes, with particular focus on the subcellular compartment where the signaling events occur. The molecules discussed and their subcellular localization are summarized in Table 1. We have separated podocytes into five compartments: the apical membrane, the slit diaphragm, the basolateral membrane, the cytoplasm, and the nucleus (Figure 1). Of all the molecules discussed, only a few have been studied for their subcellular localization by high resolution images such immunogold electron microscopy or super-resolution immunofluorescence microscopy. Thus, some of the localizations have been inferred from known protein-protein interactions or the established signaling pathways. Finally, we provide our synthesized views of the current understanding and propose future research directions.

## 2. Apical Membrane

**Figure 2:**
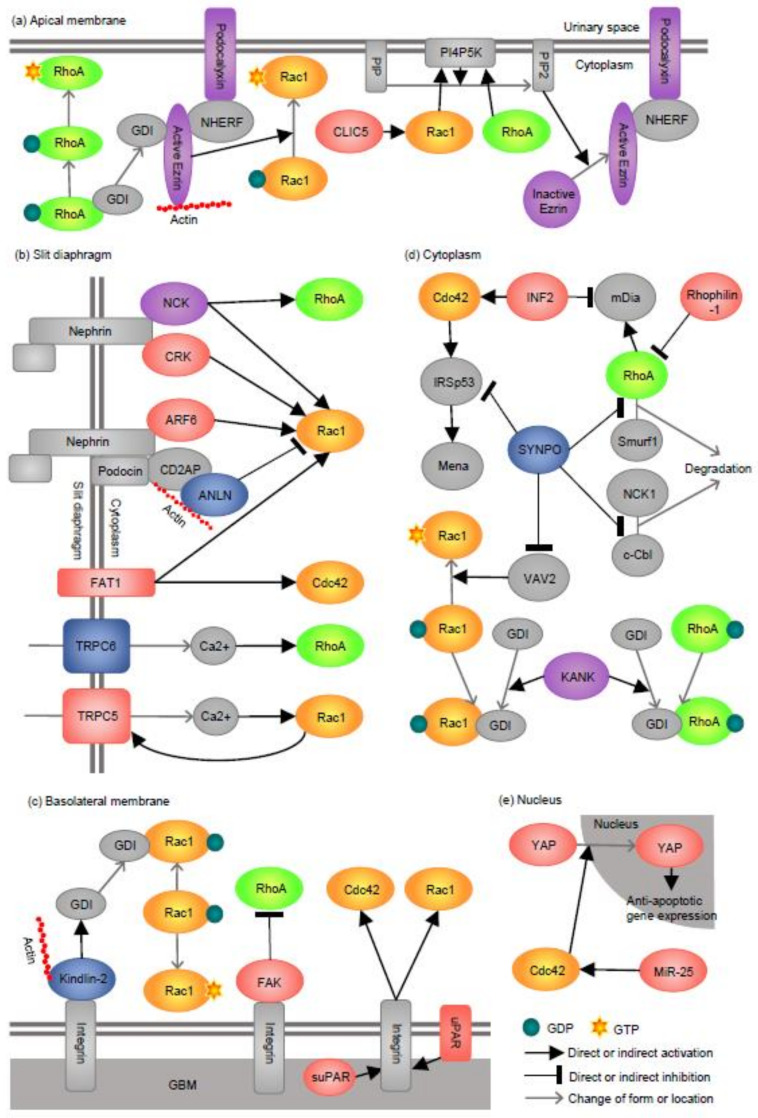
Inferred signaling events involving Rho GTPases in podocytes. Rho GTPase interacting proteins whose functions were studied in podocytes are shown by the compartment; apical membrane (**a**), slit diaphragm (**b**), basolateral membrane (**c**), cytoplasm (**d**), and nucleus (**e**). Descriptions of panel a-e are provided as short summaries at the end of Section 2, Section 3, Section 4, Section 5 and Section 6 in the text. ADP ribosylation factor 6; CD2AP: CD2 associated protein; CLIC5: Chloride intracellular channel 5; CRK: CRK proto-oncogene, adaptor protein; FAK: Focal adhesion kinase; FAT1: FAT atypical cadherin 1; GDI: GDP dissociation inhibitor; GDP: guanosine diphosphate; GTP: guanosine triphosphate; INF2: Inverted formin, FH2 and WH2 domain containing; KANK: KN motif and ankyrin repeat domains; MiR-25: microRNA-25; NCK: NCK adaptor protein; NHERF: Na^+^/H^+^ exchanger regulatory factor; PIP: Phosphatidylinositol 4-phosphate; PIP2: Phosphatidylinositol 4,5-bisphosphate; PI4P5K: phosphatidylinositol 4-phosphate 5-kinase; Smurf1: SMAD specific E3 ubiquitin protein ligase 1; SYNPO: Synaptopodin; TRPC: Transient receptor potential cation channel subfamily C member; uPAR: Urokinase plasminogen activator receptor; suPAR: soluble form of uPAR; VAV2: VAV guanine nucleotide exchange factor 2; YAP: Yes associated protein.

The apical membrane domain of podocytes is the surface facing the urinary space. It has the negative charge/anti-adhesive property that allows for foot process separation and keeps the filtration slits open, while reducing the passage of anionic proteins such as albumin [50]. Here we will discuss the role of the major components of apical protein complexes (Podocalyxin, Ezrin, and CLIC5) in maintaining the integrity of the podocyte architecture (Figure 2a), orchestrated by the Rho GTPases control over the membrane-actin cytoskeletal interface.

### 2.1. Podocalyxin

Podocalyxin is a negatively charged sialoglycoprotein that is highly expressed in podocytes. It has an N-terminal mucin-like domain, a single transmembrane domain, and a cytoplasmic tail, which contains an ezrin/radixin/moesin (ERM) binding sequence and a C-terminal PDZ domain docking site, and anchors the actin cytoskeleton through ezrin and the scaffold protein, Na^+^/H^+^ exchanger regulatory factor (NHERF) [18,51].

Systemic podocalyxin KO mice exhibit anuric kidney failure, resulting in perinatal lethality [52]. While podocalyxin heterozygous mice showed no basal phenotype, they were more susceptible to the podocyte-toxin, puromycin aminonucleoside (PAN) [53]. The loss of podocalyxin in early developmental stages has devastating impact on podocyte morphogenesis and foot process formation as shown in mice and zebrafish [52,54]. These results indicate a critical role of podocalyxin for podocyte development and function.

Several studies suggested that podocalyxin regulates the actin cytoskeleton and maintains the foot process architecture via RhoA activation. The expression of podocalyxin in Madin-Darby canine kidney (MDCK) cells induced the recruitment and sequestration of Rho GDP dissociation inhibitor (RhoGDI) to the podocalyxin/NHERF/ezrin complex, which allowed the subsequent activation of RhoA [18]. On the other hand, the phosphorylation of podocalyxin, which was increased in PAN-induced and protamine sulfate (PS)-induced rat glomerular injury models, led to decreased RhoA activity, defective localization of podocalyxin, and dissociation of the podocalyxin/NHERF/ezrin complex from actin in MDCK cells [19]. The authors speculated that phosphorylation of podocalyxin at Ser415 disrupts podocalyxin/NHERF/ezrin binding, resulting in the release of RhoGDI from ezrin and subsequent RhoA inactivation.

### 2.2. Ezrin

Ezrin is a member of the ERM protein family and acts as a cross linker between podocalyxin and cortical actin. ERM proteins contain a plasma-membrane associated N-terminal FERM domain connected by a coiled-coil structure to a C-terminal domain (C-terminal ERM-association domain C-ERMAD), which contains phosphorylation and actin binding sites. Both N- and C-terminal domains are masked by intramolecular interaction in the cytosolic inactive state [55]. The phosphorylation and subsequent activation of ERM proteins result in protein stabilization and conformation changes allowing their interactions with their partners. Rho GTPases can act upstream and downstream of ERM proteins. RhoA indirectly activates ERM proteins through phosphatidylinositol 4,5-bisphosphate (PIP2) resulting from the RhoA effector protein phosphatidylinositol 4-phosphate 5-kinase (PI4P5K) in NIH/3T3 and HeLa cells [20]. On the other hand, the N-terminal domain of ERM proteins interacts with RhoGDI and indirectly activates Rho GTPases in Swiss 3T3 cells [56].

Ezrin is highly expressed in both glomerular podocytes and proximal tubules in the mouse kidney [21]. Mice with systemic ezrin knockdown (KD) presented hypophosphatemia due to reduced phosphate reabsorption in the tubules, but no basal phenotype in glomeruli [57]. However, the mice were less susceptible to adriamycin (ADR)- and lipopolysaccharide (LPS)-induced glomerular injury [21]. Unlike in the other cellular systems as above, Rac1, not RhoA activity was dependent on ezrin abundance in mouse glomeuli and cultured podocytes. Thus, the authors speculated that loss of ezrin is protective likely through Rac1 inactivation.

### 2.3. CLIC5

Chloride intracellular channel 5 (CLIC5) co-localizes with podocalyxin/NHERF/ezrin complex at the apical domain of podocyte foot processes, as well as in the glomerular endothelial cells [22,58]. Systemic CLIC5 KO mice presented mild but significant proteinuria with shortened foot processes at 3 months of age [58,59]. Additionally, CLIC5 KO mice were more susceptible to ADR-induced and deoxycorticosterone acetate (DOCA)/salt hypertensive glomerular injury [22,58]. The results suggest that CLIC5 has a protective role in maintaining glomerular filtration barrier. Deletion of CLIC5 leads to decreased ezrin abundance and phosphorylation, and subsequent disruption of the podocalyxin/NHERF/ezrin complex in glomeruli [58,59]. CLIC5A, a predominant isoform of CLIC5 in the glomerulus, activates Rac1 in the overexpression model using COS7 cells. In addition, CLIC5A generates apical PIP2 clusters in a Rac1-dependent manner [22], which are required for ezrin phosphorylation and activation [60].

 

In summary, RhoA activation via the podocalyxin/NHERF/ezrin complex is critical in podocyte development and glomerular barrier function. Ezrin is suggested to interact with RhoGDI in podocytes, which allows the release of Rac1 and RhoA and subsequent activation. Rac1 activation via CLIC5 is important for the normal barrier function and the protection from podocyte injury. RhoA and CLIC5A-induced Rac1 stimulate PIP2 formation, which in turn activates ezrin. Overall, these pathways are required for normal podocyte function however, the overactivation of Rac1 could be detrimental in certain context [21] (Figure 2a).

## 3. Slit Diaphragm (SD)

Among the five cellular compartments in podocytes, the slit diaphragm (SD) represents the signature structure that is critical for the morphology and function of podocytes. The structural backbone of the SD is the transmembrane protein, nephrin that forms a membrane-like structure connecting adjacent foot processes via counter-parallel homotypic binding of the extracellular domain [61]. In addition, the short intracellular domain of nephrin interacts with a number of proteins and acts as the signaling hub [5,62]. In this section, we will discuss three proteins (NCK, CRK, and ARF6) that were reported to act downstream of nephrin involving Rho GTPases. An additional four proteins (ANLN, FAT1, TRPC5, and TRPC6) will be discussed in this section since the data supports their presence in the SD (Figure 2b).

### 3.1. NCK

NCK adaptor protein 1 (NCK1) and 2 (NCK2) are adaptor proteins which contain a Src homology 2 (SH2) and three SH3 domains. The SH2 domain of NCK binds to cytoplasmic phosphotyrosine residues of nephrin, while the SH3 domains interact with various effector proteins [63]. Clustering of nephrin at the cell membrane induces local recruitment of NCK and subsequent actin polymerization [63]. We also showed that nephrin stimulates cellular process formation via NCK in HEK293T cells [64] and activates Rac1 and changes morphology in cultured rat podocytes [23]. A later study demonstrated that the SH3 domain 2 of NCK1, but not of NCK2, is required for RhoA activation and downstream stress fiber formation [24], suggesting a role of nephrin-NCK1-RhoA in actin polymerization. However, NCK1 KO mice showed no glomerular phenotype, whereas when NCK2 was further deleted in podocytes, mice developed congenital nephrotic syndrome [63]. The results suggest that NCK1 and NCK2 have redundant but important roles in actin regulation and normal function in podocytes. RhoA via NCK1 likely contributes to actin regulation but other Rho GTPases such as Rac1/Cdc42 could participate since many of the NCK partner proteins via the SH3 domains, such as Pak1 and Wiskott-Aldrich syndrome protein (WASP), act downstream of Rac1/Cdc42.

It should be noted that while NCK and CRK/ARF6 discussed below have been shown to play a role in nephrin-induced signaling pathways, they may also act downstream of other membrane proteins. For example, knock-in mice of the nephrin mutant that does not interact with NCK (Y3F) showed much milder phenotype than podocyte-specific NCK1/2 KO mice [65]. This suggests that the severe phenotype of NCK1/2 KO in podocytes may be the combined effects of impaired signaling from several NCK partner proteins including nephrin.

### 3.2. CRK

Similar to NCK discussed above, CRK proto-oncogene, adaptor protein (CRK) was also found in the signaling paths downstream of nephrin. CRK1/2 and its homolog CRKL are cytoplasmic adaptor proteins and interact with nephrin [25]. Cultured podocytes with either CRK1/2 or CRKL KD showed reduced lamellipodia formation, suggesting (although not directly proven) that CRK is required for nephrin-induced Rac1 activity. Podocyte-specific deletion of either CRK1/2 or CRKL in mice was protective against PS-induced foot process effacement, and the former was also protective in nephrotoxic serum nephritis [25,26]. Importantly, podocyte-specific CRK1/2 and CRKL double KO mice showed mild (but significant) proteinuria with foot process effacement, while single CRK1/2 or CRKL KO mice presented no basal phenotype [25,26]. The results indicate that CRK1/2 and CRKL have redundant and important roles in the normal development and basal health of podocytes but can act as a pathogenic mediator in the disease context by over-activating Rac1.

### 3.3. ARF6

ADP ribosylation factor (ARF) family proteins are known to regulate vesicular trafficking and cell morphology. ARF6 interacts with nephrin and is required for nephrin-mediated Rac1 activation and membrane ruffling in cultured podocytes [27]. Podocyte-specific ARF6 KO mice presented no basal phenotype, but were protected from PS-induced injury, while showed delayed recovery from another injury model caused by nephrotoxic serum [27]. These findings suggest that ARF6 is dispensable for podocyte development and the effect of ARF6 on Rac1-mediated podocyte injury remains uncertain.

### 3.4. ANLN

Anillin actin binding protein (ANLN) is an F-actin binding protein and induces F-actin bundles at epithelial cell junctions. ANLN interacts with CD2 associated protein (CD2AP), which is an adaptor molecule linking nephrin to the actin cytoskeleton. Deletion of ANLN caused nephrotic phenotype in zebrafish, and to date, two heterozygous mutations in the ANLN gene (c.1852G>T: p.G618C and c.1291C>T: p.R431C) have been reported in familial focal segmental glomerulosclerosis (FSGS) patients [66]. Overexpression of the ANLN R431C mutant in cultured podocytes led to reduced binding affinity to CD2AP and enhanced cell motility [66]. The increased migration in R431C-overexpressing podocytes was dependent on Rac1, whereas RhoA activity was comparable [28]. Although ANLN is well known to its interact with RhoA [67], the finding suggests that ANLN likely binds to CD2AP at the SD and prevents Rac1 hyperactivation in podocytes.

### 3.5. FAT1

A member of cadherin superfamily, FAT atypical cadherin 1 (FAT1) is a transmembrane protein at the SD domain in podocytes [68]. To date, two homozygous (c.3093_3096del: p.P1032Cfs*11 and c.857A>G: p.N286S) and four compound-heterozygous (c.3008C>T: p.A1003V, c.9259C>T: p.R3087G, c.4517G>A: p.R1506H and c.5671C>A: p.P1891T) mutations in the *FAT1* gene have been reported in steroid-resistant nephrotic syndrome patients [29]. Podocyte-specific FAT1 KO mice presented massive proteinuria and glomerulosclerosis at 4 months old [29]. Depletion of FAT1 in zebrafish caused pronephric cyst (manifestation of nephrotic proteinuria in zebrafish), which was partially rescued by the Rac1/Cdc42 activator. Rac1 and Cdc42 activity were decreased in cultured podocytes with FAT1 KD, while RhoA activity remained unchanged. Similar to the zebrafish model, the Rac1/Cdc42 activator partially rescued impaired motility observed in FAT1 KD podocytes [29]. These results suggest that FAT1 facilitates Rac1/Cdc42 activity at the SD, but the mechanism was not investigated in this study.

### 3.6. TRPC5 and TRPC6

Transient receptor potential cation channel subfamily C members (TRPCs) are a family of non-selective cation channels. Since 2005, a number of TRPC6 mutations have been reported as causative for familial FSGS [69,70,71,72]. TRPC6 localizes at the SD and was shown to interact with the SD proteins, nephrin and podocin [70,73]. Most of the mutants cause gain-of-function and are expected to cause cell injury via increased calcium entry into the cell. TRPC6-mediated calcium influx was shown to induce RhoA activity, and this may also contribute to podocyte injury [30]. Another study showed that the transmembrane heparin sulfate proteoglycan, syndecan 4, suppresses the RhoA signaling pathway, thereby promoting TRPC6 abundance and calcium influx [31]. Thus, RhoA appears to be both downstream and upstream of TRPC6, however, there is no concrete evidence that RhoA plays a role in TRPC6-mediated podocyte injury.

TRPC5 also belongs to the TRPC family [30]. Unlike TRPC6, the C-terminal part of TRPC5 contains a PDZ-interacting domain, which allowed murine and rat TRPC5 to interact with NHERF if in HEK293 cells [74]. While the presence of TRPC5 in podocytes has been reported [32], its subcellular localization has not been determined definitively. TRPC5 is upregulated in proteinuric diseases and TRPC5 activation by LPS and PS evokes calcium influx, leading to synaptopodin degradation and Rac1 activation (see below) [32]. In another study, Rac1 was shown to promote the vesicular insertion of TRPC5 into the plasma membrane, suggesting a positive feedback loop between TRPC5 and Rac1 [33]. Genetic deletion or pharmacological inhibition of TRPC5 by ML204 in mice was protective in both LPS- and PS-induced models [32]. Another TRPC5 inhibitor, AC1903, suppressed proteinuria and podocyte loss in both Type-1 angiotensin II receptor transgenic and hypertensive rat models [75]. Thus, TRPC5 inhibition could be an effective therapeutic intervention in proteinuric kidney disease, and this may involve inhibiting Rac1 hyperactivation.

 

In summary, the SD proteins signaling is predominantly mediated via Rac1. The majority of the studies support the important role of Rac1 in the normal development and basal maintenance of podocyte morphology, but in certain circumstances, Rac1 hyperactivation appears to be pathogenic for example with ANLN loss or the Rac1-TRPC5 positive feedback loop. RhoA was described in association with NCK1 and TRPC6, but its role in the SD signaling is not clear (Figure 2b).

## 4. Basolateral Membrane

The basolateral membrane corresponds to the “sole” of the foot process where a number of adhesion molecules connect the foot process to the underlying GBM. Each adhesion molecule associates with numerous intracellular proteins that transmit signals to the actin cytoskeleton. When the podocytes are severely injured, they detach from the GBM and are lost in the urine. When the resulting podocyte loss reaches a critical level, the glomerulus progresses into an irreversible pathway of scarring called glomerulosclerosis [76]. Thus, the adhesion dynamics, including intracellular signaling, at the basolateral membrane is believed to have a critical role in podocyte health and disease. Readers are referred to the excellent reviews that provide extensive overview of the molecules at the basolateral membrane [3,6]. In this section, we will discuss selected proteins (FAK, Kindlin-2, uPAR, suPAR, and Integrin) that have been shown to involve Rho GTPases in their function (Figure 2c).

### 4.1. FAK

Focal adhesion kinase (FAK) is a nonreceptor tyrosine kinase and activates critical signaling pathways required for cell adhesion and motility. In podocytes, FAK resides in the protein cluster around the cytoplasmic domain of the adhesion molecules, integrins. Both podocyte-specific gene deletion and the pharmacological inhibition of FAK in mice were protective in the podocyte injury models by LPS and nephrotoxic serum [34]. In cultured podocytes, Rac1 activity was increased and RhoA activity was decreased after LPS treatment. In FAK KO podocytes, LPS activated Rac1 but the suppression of RhoA was not observed [34], suggesting that RhoA suppression, but not Rac1 activation, is dependent on FAK. LPS-induced sustained stress fibers observed in FAK KO podocytes were reversed by the Rho-kinase inhibitor [34]. Thus, although FAK is generally linked to Rac1 activation in other cell systems, in podocytes, it appears to impair the cytoskeleton by mediating stimulus-induced loss of RhoA activity. However, how RhoA activity is suppressed by FAK was not elucidated in this study.

### 4.2. Kindlin-2

Kindlin-2, a FERM domain-containing protein, is one of the cell-matrix adhesion components, which links integrins to the actin cytoskeleton. Podocyte-specific Kindlin-2 KO mice developed progressive proteinuria with foot process effacement starting at 2 weeks of age and all died by 10 weeks of age due to renal failure [35]. Cultured podocytes with Kindlin-2 KD/KO presented loss of actin stress fiber, suppressed focal adhesion formation, and increased cell motility. Rac1 activity was significantly higher in Kindlin-2 KD podocytes compared with controls, whereas RhoA activity was unchanged. Kindlin-2 interacted with RhoGDIα and loss of Kindlin-2 in podocytes promoted Rac1 release from RhoGDIα. Interestingly, the total RhoGDIα expression was also decreased in Kindlin-2 KD/KO podocytes compared with controls, but the mechanism was not described. The Rac1 inhibitor (NSC23766) partially, but not completely, restored proteinuria and the survival rate in Kindlin-2 KO mice [35]. Thus it is reasonable to conclude that the Kindlin-2, likely at the intracellular domain of integrins, suppresses hyper-activation of Rac1 via RhoGDIα, thereby maintaining stable podocyte adhesion to the GBM.

### 4.3. uPAR, suPAR, and Integrin

Urokinase plasminogen activator receptor (uPAR) is a glycosylphosphatidylinositol anchored membrane protein. Glomerular uPAR was upregulated in patients with FSGS and diabetic nephropathy, as well as rodent PAN, LPS, and lupus models [36]. Systemic deletion of uPAR in mice was protective in the LPS mouse model. Transferring uPAR gene into glomerular podocytes, but not into endothelial cells, lead to proteinuria, suggesting that uPAR in podocytes mediates LPS-induced podocyte injury. As the mechanisms, the authors demonstrated that uPAR activates αvβ3 integrins and proposed that the resulting Rac1 and Cdc42 activation leads to podocyte injury [36]. The same group later reported that the soluble form of uPAR (suPAR) may be a circulating factor that causes podocyte damage in idiopathic FSGS [77]. While this hypothesis has not been supported by independent studies, a potential role of suPAR in podocyte injury has been reported in several studies. For example, acid sphingomyelinase-like phosphodiesterase 3b (SMPDL3b), which is involved in sphingomyelin catabolism, was shown to bind to uPAR/suPAR and inhibits their ability to activate Rac1 by interfering the interaction between uPAR/suPAR and β3 integrin [37]. Thus, hyper-activation of Rac1 and possibly Cdc42 downstream of β3 integrin appears detrimental to podocyte health, consistent with the findings with Kindlin-2 as discussed above.

It is worth noting that we reported previously that activation of Rac1 in cultured podocytes reduces the cell surface expression of β1 integrin via p38 MAPK activation and this likely causes cell detachment [13]. Therefore, there may be a feedback loop between the activation of integrins and Rac1 at the basolateral membrane of podocytes that collectively leads to cell migration and detachment.

 

In summary, RhoA activity is important for stable podocyte attachment to GBM at the basolateral membrane and the activation of FAK impairs podocytes via RhoA suppression. In contrast, the activation of Rac1/Cdc42 by the loss of Kindlin-2 or by uPAR/suPAR promotes podocyte detachment thus is detrimental to podocyte health (Figure 2c).

## 5. Cytoplasm (Actin Cytoskeleton)

Most of the proteins expressed at the three membrane domains of the foot processes discussed above are linked to the intracellular actin cytoskeleton either directly or via anchor proteins. Organized actin filaments form a dynamic network in the foot process and are essential for their intricate structure whereas disruption of the actin cytoskeletal network causes foot process effacement, a common stress response to podocyte injury. Rho GTPases are one of the major regulators of actin polymerization/crosslinking and disassembly. Here, we will discuss several Rho GTPase regulators (SYNPO, INF2, KANK and Rhophilin-1), which organize the actin cytoskeleton in the cytoplasm of podocyte foot processes (Figure 2d).

### 5.1. SYNPO

Synaptopodin (SYNPO) is localized in the foot processes and a key stabilizer of podocyte actin cytoskeleton. Systemic deletion of SYNPO in mice causes delayed recovery from foot process effacement and proteinuria in PS- and LPS-induced models respectively [78]. SYNPO stabilizes RhoA from ubiquitination and subsequent proteasomal degradation by blocking the binding of the ubiquitinating enzyme, SMAD specific E3 ubiquitin protein ligase 1 (Smurf1), to RhoA [38]. SYNPO also blocks the binding of another ubiquitinating enzyme, c-Cbl, to NCK1, thereby stabilizing NCK1 expression and promoting RhoA activation [24]. Thus, SYNPO facilitates stress fiber formation in podocytes. Conversely, SYNPO suppresses filopodia by disrupting the Cdc42:IRSp53:Mena signaling pathway and SYMPO KD podocytes show aberrant non-polarized filopodia [39]. SYNPO also suppresses Rac1 activity by blocking the activation of Rac1-GEF VAV guanine nucleotide exchange factor 2 (VAV2) [40]. Thus the evidence is strong that SYMPO is critical for the actin cytoskeleton health by maintaining RhoA activity and suppressing Rac1/Cdc42.

In cultured podocytes, TRPC5-mediated calcium influx promotes SYNPO degradation and Rac1 activation [32]. Deletion of TRPC6 also promotes SYNPO degradation and this is rescued by the calcineurin inhibitor, cyclosporine A [30]. The results suggest that TRPC5 facilitates SYNPO degradation, while TRPC6 protects against it, leading to antagonistic effects on the stabilization of actin cytoskeleton.

### 5.2. INF2

Inverted formin, FH2 and WH2 domain containing (INF2) is a member of the formin family proteins. Formins are involved in actin polymerization and the *INF2* gene mutation is a common cause of familial (autosomal dominant) FSGS with/without Charcot-Marie-Tooth disease. INF2 contains an N-terminal diaphanous inhibitory domain (DID), formin homology 1 and 2 (FH1 and FH2), and C-terminal diaphanous autoregulatory domain (DAD). To date, all pathogenic FSGS mutations of INF2 have been identified in the DID [79,80]. INF2 is cleaved into two fragments by cathepsin proteases, and this allows the N-terminal DID to translocate to the cell membrane. Then, the DID of INF2 promotes cell spreading in podocyte foot processes by negatively regulating RhoA/mDia (mammalian homolog of Diaphanous) signaling [41]. INF2 KD in cultured podocytes leads to a shift in mDia to cell membrane and impaired trafficking of the SD proteins (nephrin and podocin), whereas RhoA distribution and activity remain unchanged [41]. The results suggest that INF2 counteracts mDia independently of RhoA. One of the disease-associated INF2 mutants, R218Q causes mislocalization of the cleaved N-fragment in cultured podocytes [81]. Although R218Q knock-in mice appeared grossly normal, they presented delayed recovery of foot process effacement by heparin sulfate in PS-induced injury model [82]. The precise localization of INF2 in podocytes is yet to be determined.

On the other hand, INF2 interacts with Cdc42 [83]. Many of the disease-causing INF2 mutants (R106P, L165P, and R218Q) enhance INF2-active Cdc42 interaction in HEK293 cells and inhibits translocation of Cdc42 to the plasma membrane in HeLa cells [80]. However, the interaction was decreased in podocytes transfected with the INF2 mutant, S85W, while unchanged with another mutant, S129_Q130ins [42]. Thus, the affinity with Cdc42 may be dependent on the mutants.

### 5.3. KANK

KN motif and ankyrin repeat domains (KANK) protein controls actin polymerization and is predominantly localized at the cytoplasm [84]. To date, two recessive homozygous mutations of *KANK2* gene (c.541A>G: p.S181G and c.2051C>T, p.S684F) have been reported in nephrotic syndrome patients [43]. Both dKANK (unique KANK family protein in Drosophila) KD in Drosophila cardiac nephrocyte and KANK2 KD in zebrafish led to nephrotic phenotype. Cultured podocytes with KANK2 KD presented increased RhoA activity with decreased migration, while Rac1 and Cdc42 activities remained unchanged. Both increased RhoA activity and decreased migration were rescued by overexpression of wild-type KANK2 but not by KANK2 variants, suggesting loss of function in the variants. KANK2 interacts with RhoGDIα. One of the missense KANK2 variants showed higher binding affinity of RhoGDIα to RhoA, Rac1, and Cdc42 compared with wild-type KANK2 in cultured podocytes. Similarly, KANK1 KD in cultured podocytes also presented decreased migration rate with increased RhoA and Rac1 activity, whereas Cdc42 activity was unchanged [43]. The results suggest that KANK suppresses Rho GTPase, predominantly RhoA, activity via RhoGDIa, but additional experiments are needed to validate that the signal is dependent on RhoA.

### 5.4. Rhophilin-1

Rhophilin-1 is known to interact with RhoA in a yeast two-hybrid system [85]. Systemic Rhophilin-1 KO mice developed proteinuria starting at 2 weeks old [44]. Primary cultured podocytes from KO mice presented ventral stress fibers with less peripheral actin network compared with those from control mice. Rhophilin-1 overexpression in cultured human podocytes caused a reduction in stress fiber formation and in downstream myosin II light chain phosphorylation. While RhoA activity was not shown directly, the result suggests that Rhophilin-1 has an inhibitory effect on RhoA signaling pathway. However, the deletion of RhoA in the Rhophilin-1 KO mice exacerbated the phenotype thus the role of RhoA in the phenotype of Rhophilin-1 KO mice is inconclusive.

 

In summary, SYNPO plays important roles in the maintenance of the actin cytoskeleton by stabilizing RhoA, while suppressing Rac1/Cdc42 hyperactivation. RhoA stabilization by SYNPO is critical for the recovery after podocyte injury. INF2, KANK, and Rhophilin-1 appear to protect podocytes from overactivation of the RhoA/mDia signaling pathway under basal conditions. INF2 also interacts with Cdc42 and many disease-causing mutants appear to have the increased affinity to Cdc42, thereby inhibiting its translocation to the membrane (Figure 2d).

## 6. Nucleus

Although Rho GTPases in general are known to play important roles in cell survival, proliferation, and transcriptional regulations, to date only a few reports are available studying their roles in the nucleus of podocytes (Figure 2e). The best described is in association with the transcriptional regulator, Yes associated protein (YAP) [45,46,86]. YAP is a transcriptional co-activator of the Hippo signaling pathway. Dephosphorylation of YAP promotes its translocation to the nucleus and upregulates anti-apoptotic gene expression. Podocyte-specific YAP KO mice presented progressive proteinuria starting at 5–6 weeks old and podocyte apoptosis [86]. Deletion of Cdc42 in mesenchymal progenitor cells increased cytoplasmic YAP and reduced YAP-dependent gene expression [45]. We recently showed that loss of ARHGEF7 in podocytes caused cell apoptosis due to decreased Cdc42 activity and subsequent cytoplasmic retention of YAP [46]. These results suggest that Cdc42 is required for YAP translocation to the nucleus.

Another support for the importance of Cdc42 in podocyte health can be found in the study by Liu et al. [47], which showed that microRNA-25 (**MiR-25**) is important in the expression of the Ras-signaling related genes including Cdc42. MiR-25 was downregulated in diabetic rodent model, and overexpression of miR-25 agomir injection alleviated proteinuria. Conversely, inhibition of miR-25 by antagomir injection in normal mice caused proteinuria [47]. Thus, some of the protective effect of MiR-25 may be via maintaining Cdc42 activity in podocytes.

 

In summary, a limited number of studies indicate that Cdc42 activity is critical for podocyte survival through the transcriptional regulation of anti-apoptotic/pro-survival genes via YAP. MiR-25 may facilitate podocyte survival, in part by upregulating Cdc42 expression (Figure 2e).

## 7. Rho GTPase Interacting Proteins Whose Localization Could Not Be Identified

There are several proteins that have been shown to play important roles in podocyte health involving Rho GTPases, while their precise localization within podocytes has not been clearly documented. In this section, we will discuss such proteins (**aPKC** and **NMDAR1)**.

### 7.1. aPKC

Atypical protein kinase C (aPKC) regulates cell polarity with Par proteins. Podocyte-specific aPKCλ/ι KO mice presented congenital nephrotic syndrome [87]. RhoA, Rac1, and Cdc42 activity were all increased in aPKCλ/ι KO cultured podocytes [48]. mRNA expression of Def-6, which is known to be a GEF for Rac1 and Cdc42 in other cells, was upregulated in the aPKCλ/ι KO mouse glomerulus and cultured podocytes. Furthermore, membrane-associated Def-6 was increased in aPKCλ/ι KO cultured podocytes [48]. While the authors speculated that the phenotype of aPKCλ/ι KO was due to increased Def-6 activity, direct Rho GTPase regulation by Def-6 in podocytes needs to be investigated.

### 7.2. NMDAR1

N-methyl-D-aspartate receptors (NMDARs) are widely expressed ionotropic glutamate receptors. One of the functional subunits of NMDARs, NR1, was upregulated in the glomerulus of diabetic patients and mice, and cultured podocytes treated with high glucose [49]. NR1 blockade by retrograde ureteral shRNA delivery attenuated proteinuria and foot process effacement in a diabetic mouse model [49]. Cdc42 activity and filopodia formation after treatment with high glucose were significantly increased in NR1 KD podocytes [49]. This suggests that NR1 suppresses Cdc42 activity and participates in diabetic podocyte injury.

## 8. Summary and Future Directions

It is evident that Rho GTPases interact with multiple proteins in podocytes and transduce signals from the cell membrane to the sub-membranous actin cytoskeleton or in the cytoplasm and the nucleus. While the existing data are in conflict at times, we have provided at the end of each section our synthetic views on the roles that Rho GTPases play in each subcellular domain. Overall, we believe the following:

1. In the apical membrane and the SD, a certain level of RhoA and Rac1 activities are important for the normal development and basal maintenance of podocyte morphology.
2. In all three membrane domains of foot processes, overactivation of Rac1/Cdc42 is pathogenic and contributes to podocyte injury.
3. In the basolateral membrane and the cytoplasm, RhoA abundance/activity correlates with rapid recovery after podocyte injury.
4. A certain level of Cdc42 activity is critical for podocyte survival because it is required for YAP translocation to the nucleus and subsequent expression of anti-apoptotic genes.

Considering the above, several strategies can be proposed to protect podocytes and reduce proteinuria. First is to inhibit the Rac1 pathway. The accumulated evidence collectively supports the contention that the hyperactivation of Rac1, particularly at the basolateral membrane, is pathogenic. However, Rac1 is ubiquitously expressed and plays important roles in many fundamental cellular functions. Thus, rather than inhibiting the Rac1 activity directly, targeting other molecules in the Rac1 pathway that are relevant in podocytes could be a better strategy. In this context, it is noteworthy that TRPC5 inhibition has been shown to protect podocytes likely via mitigating the downstream Rac1 activation [32,75]. It has been also shown that aberrant Rac1 signaling causes the mineralocorticoid receptor activation in podocytes, thus mineralocorticoid receptor antagonists can be viewed as the inhibitor of the Rac pathway [88]. Stabilization of integrin at the basolateral membrane and/or blocking integrin endocytosis may also be a promising way to antagonize the Rac1 effect at the basolateral membrane and to prevent podocyte detachment from the GBM. Second is to stabilize RhoA and actin filaments. For the last few decades, calcineurin inhibitors have been used in the treatment of nephrotic syndrome. In addition to their known immunosuppressive properties on T cells, they prevent synaptopodin degradation, thereby maintaining RhoA stability in podocytes [89]. Thus, other drugs that can directly or indirectly maintain the RhoA activity in podocytes will be likely therapeutic in proteinuria. Alternatively, recent studies showed that Bis-T-23 that promotes oligomerization of large GTPase dynamin was protective in injury models by stabilizing actin filaments [90]. Thus, direct stabilization of actin filaments is another possibility. Finally, enhancing the Cdc42-YAP pathway can be considered as another way to prevent podocyte apoptosis.

It should be noted that our knowledge is limited regarding the regulatory proteins that directly control the activities of Rho GTPases and the interacting proteins that mediate cellular events in podocytes. With the goal of establishing the protein network of Rho GTPases, their regulatory proteins and the interactors in podocytes, we are currently conducting a proximity-based ligation assay (BioID) [91] and proteomics analyses using a series of Rho GTPases and their mutants as bait. Furthermore, the studies performed so far have been focused on the three prototypical Rho GTPases (RhoA, Rac1, and Cdc42), but very little is known about the role of the remaining 17 Rho GTPases. An additional knowledge gap is the temporal and spatial activities of Rho GTPase pathways in podocytes. Such studies will require three-dimensional in vitro system. We believe that utilizing induced pluripotent stem (iPS) cell-derived kidney organoids or podocytes could be useful to better define where and when Rho GTPase pathways are activated within podocytes, what other molecules are involved, and how they collectively contribute to the pathophysiology of proteinuric kidney diseases.

## Figures and Tables

**Table 1:** Summary of the Rho GTPase interacting proteins in podocytes according to their subcellular localization, corresponding Rho GTPase(s) and known modulators. MiR-25 is included, although it is not a protein, as discussed in the text.

		Rho GTPase				
Compartment	Interacting Protein	RhoA	Rac1	Cdc42	Known Modulators	Reference Number
Apical membrane	Podocalyxin	✓			Ezrin, NHERF, RhoGDI	[18,19]
	Ezrin	✓	✓		RhoGDI	[20,21]
	CLIC5		✓			[22]
Slit diaphragm	NCK	✓	✓		Nephrin	[23,24]
	CRK		✓		Nephrin	[25,26]
	ARF6		✓		Nephrin	[27]
	ANLN		✓		CD2AP	[28]
	FAT1		✓	✓		[29]
	TRPC6	✓				[30,31]
	TRPC5		✓			[32,33]
Basolateral membrane	FAK	✓			Integrin	[34]
	Kindlin-2		✓		RhoGDI, Integrin	[35]
	uPAR, suPAR		✓	✓	Integrin	[36,37]
Cytoplasm	SYNPO	✓	✓	✓	VAV2, IRSp53, Smurf1, c-Cbl	[24,38,39,40]
	INF2	✓		✓	mDia	[41,42]
	KANK	✓	✓		RhoGDI	[43]
	Rhophilin-1	✓				[44]
Nucleus	YAP			✓		[45,46]
	MiR-25			✓		[47]
Undetermined	aPKC	✓	✓	✓	Def-6	[48]
	NMDAR1			✓		[49]
